# Cross-cultural consistency of image memorability

**DOI:** 10.1038/s41598-023-39988-5

**Published:** 2023-08-05

**Authors:** Su Keun Jeong

**Affiliations:** Department of Psychology, Chungbuk National University, Chungdae-ro 1, Seowon-gu, Cheongju, Chungbuk 28644 Korea

**Keywords:** Psychology, Human behaviour

## Abstract

Memorability refers to the intrinsic property of an image that determines how well it is remembered or forgotten. Recent studies have found that memorability is highly consistent across individuals. However, most studies on memorability were conducted with participants from Western cultures, and the images used in memorability studies were culturally biased. Previous studies implicitly assumed that memorability would be held constant across different cultural groups; however, to the best of our knowledge, this has not yet been empirically investigated. In the current study, we recruited participants from South Korea and the US and examined whether image memorability was consistent across these two cultures. We found that South Korean participants showed greater memory performance for images rated highly memorable by US participants. The current findings provide converging evidence that image memorability is not fully accounted for by individual differences, and suggest the possibility of cross-cultural consistency in image memorability.

## Introduction

People remember some images better than others. Factors that may influence memory performance include the personal relevance of the stimulus, emotional content of the stimulus, novelty or uniqueness of the stimulus, and degree to which the stimulus stands out from other competing stimuli^1–7^. Recent studies further suggest that stimuli have intrinsic memorability that cannot be fully explained by previously well-known image properties, such as perceptual distinctiveness, interestingness, aesthetics, or semantic features^8–12^. Consistent memorability has been found in visual stimuli such as faces and scenes^8,12^, auditory stimuli^13^, dynamic stimuli such as movies of naturalistic scenes and human actions^14,15^. In addition, different top-down encoding strategies do not significantly affect stimulus memorability^16,17^. Neither low-level visual features (e.g., color or pixel intensity) nor high-level semantics (e.g., object contents or aesthetics) alone successfully predicted stimulus memorability, suggesting that memorability could explain the unique variance in memory performance^8,9,18,19^.

Previous studies have argued that intrinsic image memorability is highly consistent across participants, and is not affected by individual differences. In other words, if a person can remember a certain image well, another individual is also likely to remember the image well. Most studies on memorability have been conducted with participants from Western countries. In addition, the stimuli that were tested for memorability were culturally biased. One of the most widely used image databases for testing memorability is the 10k US Adult Face Database^8^. This database contains faces whose demographics match the US population. The memorability scores of the face images were measured based on the memory performance of Amazon Mechanical Turk (MTurk) workers whose demographics also matched the US population. In contrast, the MemCat image database contains a diverse range of scene categories and thus may be culturally less biased than the 10k US Adult Face Database^20^. However, the memorability scores of the MemCat images were obtained mostly from MTurk workers who indicated that they were living in the US. Therefore, although studies on image memorability have shown highly consistent results, it is not yet clear whether such consistency can be replicated in culturally different groups of participants as either existing images in the memorability database or participants who rated memorability scores were biased.

One might argue that image memorability is culture-dependent because evidence suggests that cultural differences can affect visual cognition in several ways. Research has shown that cultures may affect how people attend to and interpret visual stimuli, and cultural differences may also influence how people remember visual information. For example, people from Western cultures tend to remember more specific details of an image, whereas people from Eastern cultures tend to remember the overall context and relationships between objects^21,22^. In addition, people are better at recognizing faces of their own race compared to faces of other races^23,24^, raising the question of the cross-cultural consistency of stimulus memorability.

Nevertheless, recent findings on stimulus memorability suggest the possibility that memorability is cross-culturally consistent. In a recent study, the semantic contents of scene images were eliminated by phase-scrambling^25^. Although the meanings of the images were not recognizable, observers consistently remembered certain images better than others. This result suggests that image memorability is driven by higher-order perceptual properties, which are less likely to be culture-dependent. Interestingly, image memorability scores estimated by a convolutional neural network (CNN) designed to predict human memory performance correlated with monkey’s performances^26^. The consistency of image memorability across species further supports the possibility of cross-cultural consistency of image memorability. Nevertheless, to the best of our knowledge, no empirical study has been conducted to directly assess image memorability across different human cultural groups.

To this end, we asked participants from different cultural backgrounds to remember visual stimuli, and then compared their performances to test the cross-cultural consistency of image memorability (see Supplementary Table 1). In Experiments 1A and 1B, South Korean participants were asked to remember faces and scenes selected from the 10k US Adult Face and MemCat databases^8,20^. South Koreans’ recognition accuracies were compared with memorability scores obtained from MTurk workers in previous studies^8,20^, whose demographics were similar to those of the US population. However, the experimental paradigm used in Experiments 1A and 1B was not the same as that was used in the previous studies. Therefore, to generalize and replicate Experiments 1A and 1B, we recruited both South Korean and US MTurk workers in Experiments 2A and 2B and tested them on the same face and scene memory task. Thereafter, South Koreans’ recognition accuracy was compared with that of MTurk workers.

## Results

### Experiments 1A and 1B (Korean)

**Figure 1:**
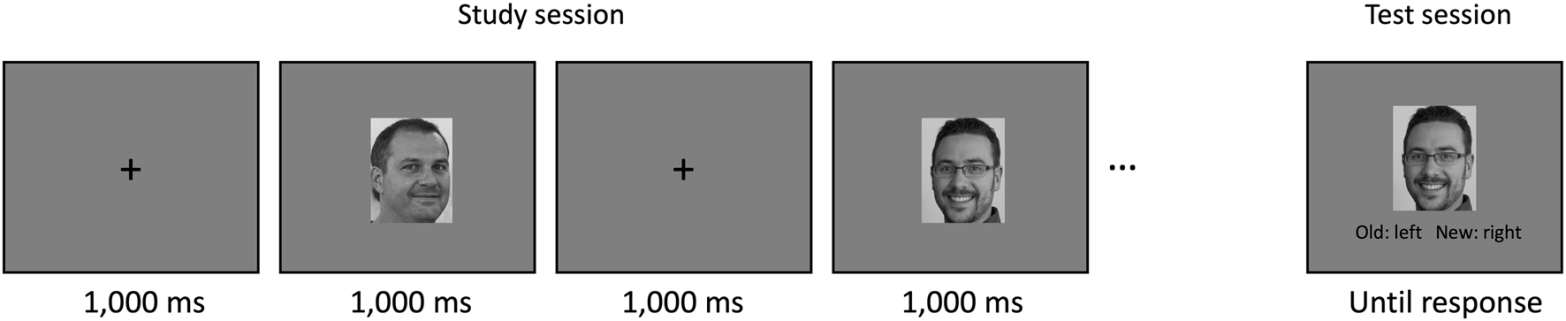
Experimental procedure of Experiments 1A and 1B. Participants were asked to remember faces during the study session. They were also required to respond when an upside-down face was shown. After the study session, a 30-s break was provided. In the test session, an old/new recognition task was used. Face images in this figure were generated using a random face generator (https://thispersondoesnotexist.com/). In Experiment 1B, scene stimuli were used in the same experimental paradigm.

**Figure 2:**
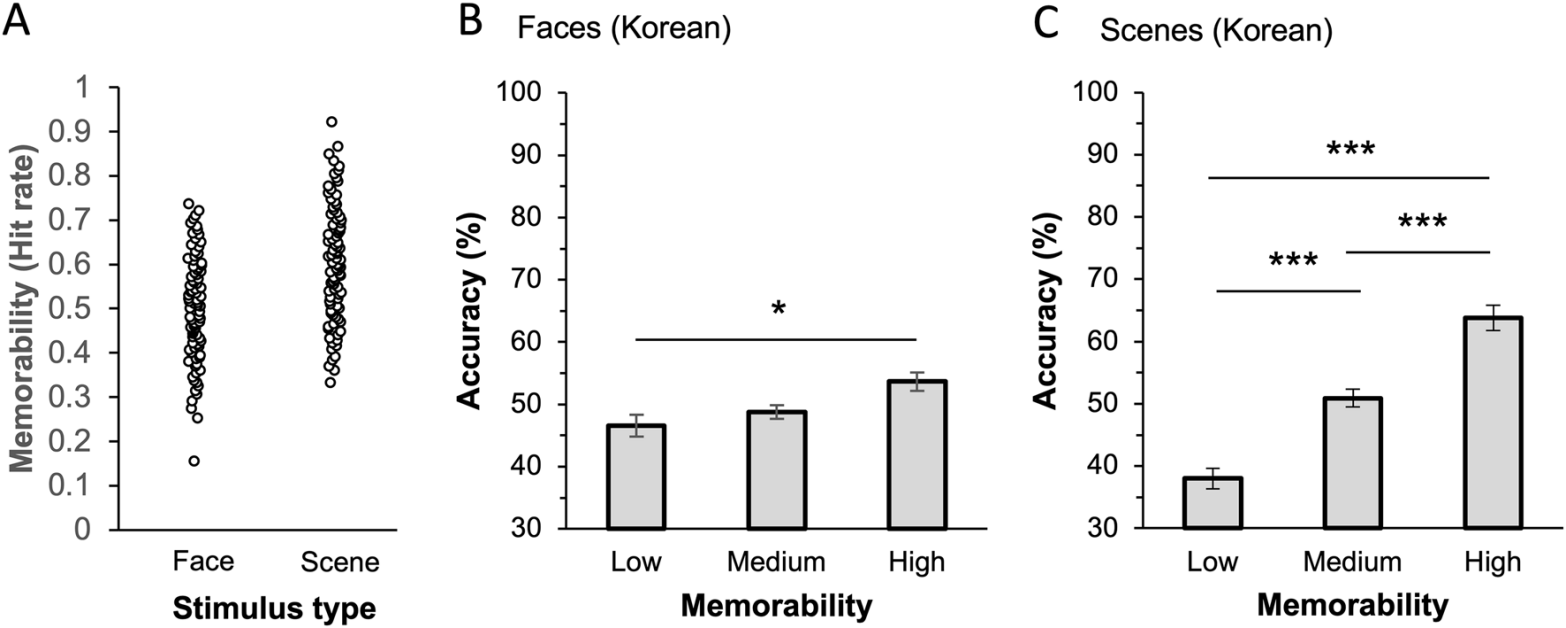
Results of Experiments 1A and 1B. (**A**) Distribution of memorability score (hit rates) of face and scene images reported in the previous studies. (**B**) South Korean participants’ recognition accuracy for low, medium, and high memorability face images. Face images were assigned to low, medium, and high memorability conditions based on their memorability score (i.e., hit rates) reported in the previous study. Results showed that high memorability face images were remembered better than low memorability faces. (**C**) South Korean participants’ recognition accuracy for low, medium, and high memorability scene images. Results showed that high memorability scenes were remembered better than both medium and low memorability scenes, and medium memorability scenes were remembered better than low memorability scenes. Error bars represent within-subject standard errors. * *p* < .05, *** *p* < .001.

In Experiments 1A and 1B, independent groups of South Korean participants were recruited and were asked to remember faces and scenes in separate experiments. In the study session, 90 faces and 90 scenes were presented for 1000 ms in Experiments 1A and 1B, respectively (Fig. 1). A recognition task was followed by a 30-s break. The face and scene stimuli were divided into high, medium, and low memorability conditions according to their memorability scores reported in previous studies^8,20^ (20, 50, and 20 images in the high, medium, and low memorability conditions, respectively; Fig. 2A shows the distribution of memorability scores of stimuli).

In Experiment 1A, a repeated-measures ANOVA was performed to compare the effect of memorability (low, medium, and high) on face recognition accuracy, and a significant effect of memorability was found, F(2, 46) = 4.05, *p* = 0.024, ${\eta }_{p}^{2}$ = 0.150 (Fig. 2B). Post-hoc analyses revealed that faces with high-memorability faces were better remembered than faces with low-memorability faces, t(23) = 2.78, *p* = 0.024, d = 0.47. However, there was no significant difference between the low and medium conditions, t(23) = 0.86, *p* > 0.99, d = 0.15, and between the medium and high conditions, t(23) = 1.92, *p* = 0.183, d = 0.33.

Experiment 1B, which used scene images, showed similar results to those of Experiment 1A. A significant main effect of memorability was observed, F(2, 48) = 36.96, *p* < 0.001, ${\eta }_{p}^{2}$ = 0.606 (Fig. 2C). High-memorability scenes were better remembered than both medium- and low-memorability scenes: t(24) = 8.60, *p* < 0.001, d = 1.33 and t(24) = 4.31, *p* < 0.001, d = 0.67, respectively. In addition, medium-memorability scenes showed higher recognition accuracy than low-memorability scenes, t(24) = 4.29, *p* < 0.001, d = 0.67. Experiments 1A and 1B used different stimulus categories but overall results were similar (see Supplementary Results for comparison between Experiments 1A and 1B).

**Figure 3:**
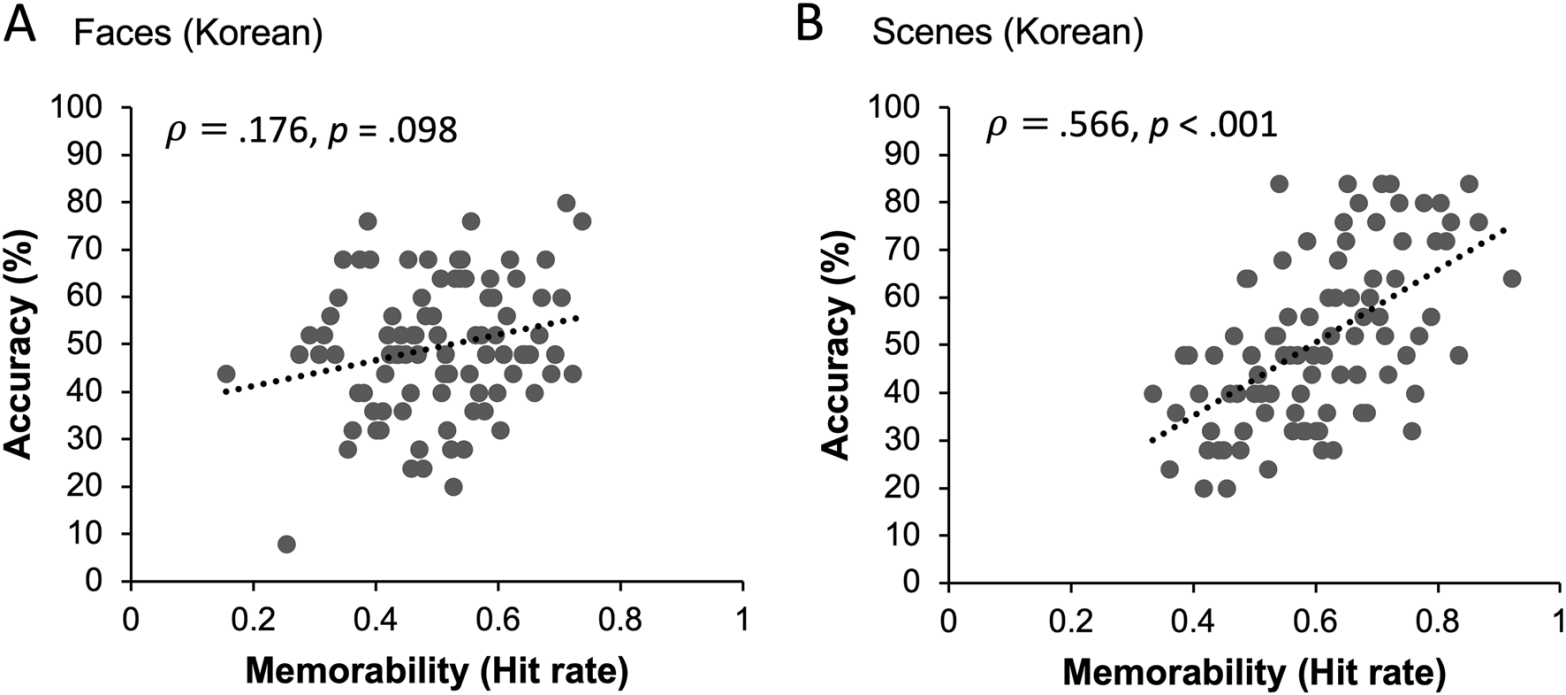
Results from Experiments 1A and 1B. (**A**) Y-axis indicates South Korean participants’ face recognition accuracy and X-axis indicates the memorability score (hit rate) of US MTurk workers reported in the previous study. Each circle represents an individual face image. South Korean participants’ memory performance showed a marginally significant correlation with US MTurk workers’ performance. (**B**) South Korean’s scene recognition accuracy for individual images was significantly correlated with the memorability score (hit rate) of US MTurk workers in the previous study. Each circle represents an individual scene image.

We divided face and scene images into low, medium, and high memorability conditions based on their hit rates provided by the MTurk workers in previous studies^8,20^. However, this division can be arbitrary or biased. Thus, to compare Korean participants’ and the MTurk workers’ memory performance objectively, we calculated Spearman’s correlation between Korean participants’ recognition accuracy for individual images obtained in the current study and the MTurk workers’ hit rates for the same images reported in the previous studies. There was a marginally significant correlation between the face memorability (hit rates of MTurk workers) and recognition accuracy of South Korean participants, $\rho$ = 0.176, *p* = 0.098 (Fig. 3A). The scene stimulus showed a significant correlation between memorability and participants’ performance, $\rho$ = 0.566, *p* < 0.001 (Fig. 3B).

The memorability scores (hit rate) of the face and scene stimuli were obtained mostly from the MTurk workers, whose demographics were similar to those of the US population. Nevertheless, South Korean participants remembered high-memorability images better than low-memorability images, suggesting that stimulus memorability remained consistent across participants from different cultural backgrounds. One might argue that the result of Experiment 1A does not strongly suggest the cross-cultural consistency of image memorability for face stimuli because the correlation between memorability score and Korean participants’ recognition accuracy was not statistically significant. However, in Experiment 1A, Korean participants’ face recognition accuracy was relatively low. If Korean participants properly encoded face images, then their memory performance could correlate with memorability scores. We tested this possibility in the follow-up experiments.

### Experiment 2A (Korean)

The overall recognition accuracy was not high in Experiments 1A and 1B. Different participants took part in the face and scene memory experiments, and thus, it may not be appropriate to directly compare face and scene memory performances. In addition, the experimental paradigm used to build the memorability database in previous studies was different from the current experimental paradigm, further complicating the comparison between memorability in current and previous studies^8,20^.

While Experiments 1A and 1B compared South Koreans’ memory performance to the memorability scores recorded in the existing database, Experiments 2A and 2B recruited both Korean and US MTurk participants and asked both groups to perform the same task. In Experiment 2A, South Korean participants were asked to perform both face and scene memory tasks; the order of the tasks was counterbalanced across participants. In Experiment 2B, MTurk workers in the US were recruited and they were asked to perform the same task. As the overall recognition performance was low in Experiments 1A and 1B, we made the task easier in Experiments 2A and 2B (see “Methods” section).

**Figure 4:**
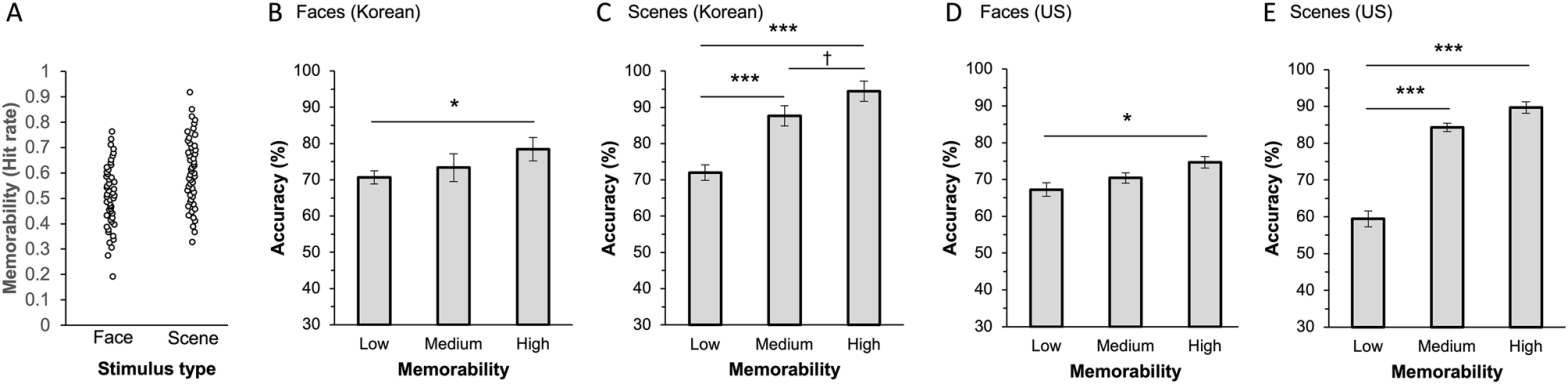
Results of Experiments 2A and 2B. (**A**) Distribution of memorability score (hit rates) of face and scene images reported in the previous studies. (**B**) and (**C**) South Korean participants’ recognition accuracy for low, medium, and high memorability faces and scenes. High memorability stimuli were remembered better than low memorability stimuli. (**D**) and (**E**) MTurk participants in the US were recruited and asked to perform the same face and scene memory tasks as South Korean participants did. In the US MTurk results, high memorability images were also remembered better than low memorability images. Error bars represent within-subject standard errors. † < .1, * *p* < .05, *** *p* < .001.

In Experiment 2A, the main effects of memorability were found for both face and scene stimuli, F(2, 58) = 3.64, p = 0.033, ${\eta }_{p}^{2}$ = 0.111, and F(1.39, 40.30) = 33.47, *p* < 0.001, ${\eta }_{p}^{2}$ = 0.536 (Fig. 4B and C). For face stimuli, high-memorability faces showed greater accuracy than low-memorability faces, t(29) = 2.65, *p* = 0.031. d = 0.50. However, no difference was found between low- and medium-memorability faces, t(29) = 0.91, *p* = 1, d = 0.17, and between medium- and high-memorability faces, t(29) = 1.74, *p* = 0.259, d = 0.33. For scene stimuli, low-memorability scenes were less remembered than both high- and medium-memorability scenes, t(29) = 7.98, *p* < 0.001, d = 1.53 and t(29) = 5.57, *p* < 0.001, d = 1.07, respectively. Further, there was a marginally significant difference between the medium- and high-memorability scenes, t(29) = − 2.41, *p* = 0.058, d = 0.46 (see Supplementary Results for comparison between face and scene experiments).

**Figure 5:**
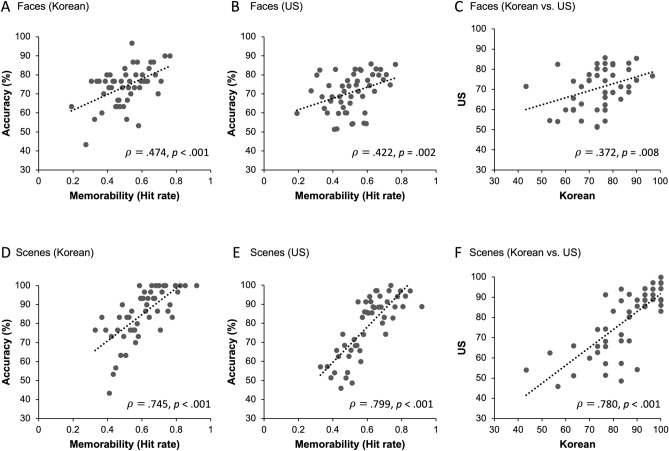
Results from Experiments 2A and 2B. (**A**) and (**D**) The memorability score of individual images (hit rate) reported in the previous study was compared to the recognition accuracy of individual images obtained in the current study. Korean participants’ recognition accuracies for face and scene images were correlated with the memorability scores of those images. (**B**) and (**E**) US MTurk worker’s recognition accuracies for face and scene images reported in the previous studies were also correlated with the memorability scores of the images obtained in the current study. (**C**) and (**F**) South Korean participants’ recognition accuracy for individual images was compared to that of US MTurk workers. Face and scene images that Korean participants remembered better were also recognized better by US MTurk workers, showing cultural consistency of image memorability.

When Koreans’ recognition accuracy for individual images in Experiment 2A and the memorability scores for the same images from previous studies were compared, a significant correlation was observed for both face and scene stimuli (faces: $\rho$ = 0.474, *p* < 0.001, scenes: $\rho$ = 0.745, *p* < 0.001; see Fig. 5A and D).

These results replicated those of Experiments 1A and 1B and provided supporting evidence of the cultural consistency of image memorability.

### Experiment 2B (US)

In Experiment 2B, the main effects of memorability were again found in both the face and scene experiments, F(2, 68) = 3.588, *p* = 0.033, ${\eta }_{p}^{2}$ = 0.095 and F(1.29, 43.80) = 63.34, *p* < 0.001, ${\eta }_{p}^{2}$ = 0.65 (Fig. 4D and E), respectively. Post-hoc analysis also revealed the same pattern of results as in Experiment 2A. For face stimuli, there was a significant difference between the low and high memorability conditions, t(34) = 2.67, *p* = 0.028, d = 0.43, but no other differences were found (low vs. medium: t(34) = 1.15, *p* = 0.767, d = 0.19; medium vs. high: t(34) = 1.52, *p* = 0.397, d = 0.25). For scene stimuli, low-memorability scenes were remembered less than both medium- and high-memorability scenes, t(34) = 8.66, *p* < 0.001, d = 1.55, and t(34) = 10.55, *p* < 0.001, d = 1.88, respectively. However, no difference was observed between the medium- and high-memorability scenes, t(34) = 1.89, *p* = 0.188, d = 0.34 (see Supplementary Results for comparison between face and scene experiments).

A comparison between individual stimulus memorability reported in a previous study and the recognition accuracy obtained from the MTurk workers in Experiment 2B showed significant correlations for both face and scene stimuli ($\rho$ = 0.422, *p* = 0.002 and $\rho$ = 0.799, *p* < 0.001; see Fig. 5B and E).

Overall, the results from the US MTurk workers in Experiment 2B were consistent with those from the Korean participants in Experiment 2A.

### Comparison between experiments 2A (Korean) and 2B (US)

Since Experiments 2A and 2B utilized the same experimental paradigm*,* we could directly compare the memory performance of South Korean and US MTurk participants. A mixed ANOVA with stimulus type and memorability as within-subject factors and participant group as a between-subject factor was conducted to compare the Korean and US MTurk participants. A significant main effect of stimulus type, F(1, 63) = 36.88, *p* < 0.001, ${\eta }_{p}^{2}$ = 0.369 was found, showing greater memory accuracy for scenes than faces on average. The main effect of memorability was also significant, F(1.65, 104.21) = 79.55, *p* < 0.001, ${\eta }_{p}^{2}$ = 0.558. Post-hoc analyses revealed that high memorability images were better remembered than medium and low memorability images, and medium memorability images were better remembered than low memorability images, ts > 3.92, ps < 0.001, ds > 0.34. Further, a marginally significant group effect was found, F(1, 63) = 3.28, *p* = 0.077, ${\eta }_{p}^{2}$ = 0.049, with Korean participants’ accuracy being numerically greater than US MTurk workers’ accuracy.

The interaction between stimulus type and memorability was significant, F(1.60, 100.61) = 24.73, *p* < 0.001, ${\eta }_{p}^{2}$ = 0.28. Post-hoc tests showed that scenes were better remembered than faces in the medium and high memorability conditions, ts > 6.28, ps < 0.001, ds > 0.88, but not in the low memorability condition, t(64) = 1.44, *p* > 0.99, d = 0.20.

Importantly, memorability and group did not show a significant interaction, F(1.65, 104.21) = 1.71, *p* = 0.191, ${\eta }_{p}^{2}$ = 0.02, suggesting that image memorability did not systemically differ between the Korean and US participants. No interaction was found between stimulus type and group, F(1, 63) = 1.48, *p* = 0.228, ${\eta }_{p}^{2}$ = 0.023, and between memorability, stimulus type, and group, F(1.60, 100.61) = 1.35, *p* = 0.262, ${\eta }_{p}^{2}$ = 0.021.

Next, we tested whether Korean participants’ recognition accuracy for individual images correlated with the MTurk participants’ accuracy for the same images. The results showed that recognition accuracy for individual stimuli was significantly correlated between the two groups of participants, demonstrating the cultural consistency of stimulus memorability (faces: $\rho$ = 0.372, *p* = 0.008, scenes: $\rho$ = 0.780, *p* < 0.001; Fig. 5C and F).

Finally, we examined whether the correlation between image memorability and recognition accuracy of Korean participants was statistically compatible with that of the US MTurk workers’ by using the TOSTER package in R. For face stimuli, the difference in correlation between Korean and the US participants was not significant, *ρ* = 0.052, *p* = 0.621, 90% CI [− 0.226–0.331]. For face stimuli, the difference in correlation was also not significant, *ρ* = − 0.055, *p* = 0.741, 90% CI [− 0.191–0.0870]. These results suggest that the image memorability was similar across the two participant groups.

## Discussion

Previous studies have shown that stimulus memorability is highly consistent among individuals^8,9,16,18^. However, studies on memorability have mostly used stimuli and employed participants from Western countries. The current study tested the cultural consistency of visual stimulus memorability by comparing participants from South Korea and the US. The results showed that Korean participants were more likely to remember face and scene images that showed higher hit rates among MTurk workers, whose demographics reflect the US population. Experiments 1A and 1B showed that Korean participants’ recognition performance was correlated with that of MTurk workers in previous studies. However, these results were not conclusive because the previous studies and the current study used different experimental paradigms. Using the same experimental paradigm between Koreans and US MTurk participants, Experiments 2A and 2B replicated the cultural consistency of memorability. To the best of our knowledge, this study is the first empirical evidence to support the consistency of stimulus memorability across cultures.

One might expect to observe worse memory performance for face images in Korean participants because the demographics of the face images resembled that of the US population and not Korea, showing the other race effect^23,24^. In the current study, no East Asian faces were used in Experiment 1A and only three East Asian faces were included in Experiment 2A. Though almost all face images were non-Asian, we did not find worse face memory performance in the South Korean participants.

The lack of the other race effect could be due to Korean participants in the current study being familiar with non-Asian faces because of frequent media exposure. Some studies have reported that the other race effect can be reduced or even reversed by training participants^27–29^; however, others have argued that frequent exposure to out-group faces did not change the other race effect^30^. A more plausible explanation for why there was no other race effect is that the East Asian faces included in the database may not look like Koreans. In other words, both East Asian and non-Asian faces in the image database may resemble foreign faces for Korean participants in the current study. Indeed, East Asian faces are not unitary, and they show substantial differences across countries^31^. Finally, because we did not ask the MTurk workers to report their race, there is a possibility that the racial composition of the MTurk workers in the current study may not reflect the demographics of the US population. Thus, further studies should be conducted to examine the relationship between the other race effect and the memorability of faces.

In the current study, we divided the study and test sessions and measured memory performance using an old/new recognition task. Previous studies that used a similar old/new memory task also showed consistent image memorability^16,32^. However, most previous studies on memorability used a continuous performance task paradigm in which participants watched a sequence of images and detected a repetition of stimuli, which occurred with different lengths of space between stimuli^8,9^. The memorability scores in the image database were also measured using a continuous performance task. Despite the differences between the experimental paradigms, the memorability (i.e., recognition accuracy) measured in the current study showed a significant correlation with that reported in image databases. Thus, the consistency of memorability was observed in different participant groups across different experimental paradigms, suggesting the possibility that the existing memorability database can be widely used to test memory in diverse settings.

It should be noted that the current results have several limitations. As mentioned above, the potential difference between in-group and out-group face images should be considered to further understand whether the other race effect interacts with intrinsic stimulus memorability. In addition, it is worth testing the cultural consistency of stimuli from other domains, such as auditory sounds, movies, and texts to generalize the current findings. Additionally, the consistency of memorability needs to be tested in different populations with different cultural backgrounds or expertise. Despite these limitations, the current findings raise an intriguing possibility that stimulus memorability is culturally universal, and further support that memorability is an intrinsic property of stimuli.

## Methods

### Participants

A previous study^17^ reported a large effect size (${\eta }_{p}^{2}$ = 0.5) when comparing memory performance for low- and high-memorability images. A power analysis using G*Power 3^33^ revealed that five participants were required when using a large effect size, $\alpha$ = 0.05, power = 0.95, in a repeated-measures ANOVA with three conditions (low, medium, and high memorability). Nevertheless, we recruited more participants to ensure sufficient statistical power. A total of 114 participants were recruited (24, 25, 30, and 35 in Experiments 1A, 1B, 2A, and 2B, respectively; Supplementary Table 1). In Experiments 1A, 1B, and 2A, South Korean participants were recruited for an offline study. In Experiment 2B, participants were recruited using MTurk. These participants were required to be US residents, have a Master’s qualification, and have a HIT approval rate greater than 98%. This study was approved by the institutional review board of Chungbuk National University (CBNU-202209-HR-0207) and all participants provided informed consent. All methods were performed in accordance with the relevant guidelines and regulations from by the institutional review board of Chungbuk National University.

### Stimuli

#### Experiments 1A and 1B

A total of 180 face and 180 scene images were selected from the 10k US Adult Faces^8^ and MemCat image databases^20^. Face images were used in Experiment 1A, and scene images were used in Experiment 1B. In each experiment, 90 images were presented as to-be-encoded items, and the remaining 90 images were used as foil items during the memory test. Based on the memory hit rate of the images reported in the database, 20, 50, and 20 images were assigned to the low, medium, and high memorability conditions, respectively, in the study session. Figure 2A shows the distribution of memorability scores of the face and scene stimuli. The memorability of the foil items was matched with that of the to-be-encoded items. A new set of 16 images was also selected from each database for the practice sessions.

#### Experiments 2A and 2B

Face and scene images were extracted from the same image databases used in Experiments 1A and 1B. A total of 100 face and 100 scene images were used. Among the 100 images, 50 were used as to-be-encoded items, and the remaining 50 images were used as foil items in the test session. Based on the hit rate of the images, 15, 20, and 15 images were labelled as low, medium, and high memorability conditions, respectively, in the study session. Figure 4A shows the distribution of memorability scores of the face and scene stimuli. A new set of 30 images was selected from each database for each practice session.

### Experimental design

The experiment was programmed using PsychoPy software^34^. Experiments 1A, 1B, and 2A were conducted offline. Each experiment was performed with different groups of South Korean participants. Experiment 2B was hosted on Pavlovia (https://pavlovia.org), involving the MTurk workers living in the US.

#### Experiments 1A and 1B

In the study session, participants were asked to remember faces (Experiment 1A) or scenes (Experiment 2B) presented at the center of the display (Fig. 1). The images were presented on a 24-inch LCD monitor and subtended at 9° × 9°. Each image was presented for 1000 ms following a 1000 ms fixation. While encoding the images, the participants were required to press the spacebar when they detected an upside-down image. A detection task was employed to ensure participants' attention to the images. Eighteen upside-down images were presented during the study sessions. After the study session, a 30-s break was provided. In the test session, participants performed an old-new recognition memory task. Half of the stimuli were encoded by participants in the study session, and the other half were novel stimuli. The test stimulus was presented in the same size as that in the study session and remained on the display until the participants responded.

A practice session was conducted before the main experiment. The practice session was the same as the main experiment except that there was no recognition task. Twelve memory images and 4 upside-down images were used in the practice session. No feedback was provided in either the practice session or the main experiment.

#### Experiments 2A and 2B

The overall experimental design was the same as that of Experiments 1A and 1B, except for the following: As Experiment 2B was conducted online, we could not precisely control for the stimulus size. Thus, in Experiments 2A and 2B, images were presented in the center and were set to subtend 35% of the screen height.

In the study session, to reduce task difficulty and make participants focus on the encoding task, the upside-down image detection task was not used. Further, to ensure sufficient encoding time, images remained on the display until the participants pressed the spacebar. To reduce the duration of the experiment and engage online participants’ attention, the delay between the images was reduced to 500 ms. A practice session that contained 15 encoding and 30 recognition test trials was performed before the main experiment. A feedback message was provided in the practice session, but no feedback was provided in the main experiment.

### Data analysis

In Experiments 1A and 1B, a repeated-measures ANOVA was performed to compare the recognition accuracy of the low, medium, and high memorability images. A Greenhouse–Geisser correction was applied when the sphericity assumption was violated. All post-hoc analyses were corrected for multiple comparisons using the Bonferroni method (two-tailed). We then calculated Spearman’s correlation between the recognition accuracy in the current study and the memorability scores (hit rates) of each image reported in the database.

In Experiments 2A and 2B, the same analyses as in Experiments 1A and 2B were conducted. Additionally, we calculated Spearman’s correlation between the recognition accuracies of the South Korean and MTurk participants. We also tested whether Korean and the US participant groups were statistically compatible by comparing the correlation coefficients between the participants groups. We used the *boot_compare_cor* function in the TOSTER package in R, with an alpha level of 0.05 and 10,000 bootstrap replicates. The *boot_compare_cor* function was designed to compare two correlations between two variables between two groups with bootstrapping. Using this function, we compared correlation between image memorability and recognition accuracy of Korean participants was equivalent with the correlation of the US MTurk workers’. A higher *p* value indicates higher compatibility between the studies.

### Supplementary Information

Supplementary Information.

## Data Availability

The data and materials for all experiments are available from the corresponding author upon reasonable request.
