# Supplementary material for: Cross-cultural consistency of image memorability
Source: Sci Rep. 2023 Aug 5;13:12737. doi: 10.1038/s41598-023-39988-5 (PMC10404227; doi:10.1038/s41598-023-39988-5)
Supplement: Supplementary file 1 — Supplementary Information. [file 41598_2023_39988_MOESM1_ESM.docx]

**Supplementary Results**

**Experiments 1A (Korean, faces) and 1B (Korean, scenes)**

To compare face and scene experiments, a mixed ANOVA with experiment (stimulus type) as a between-subject factor and memorability as a within-subject factor was conducted.

The results showed a significant effect of memorability, *F*(1.76, 82.58) = 34.69, *p* < .001, $\eta_{p}^{2}$= .425. Post-hoc analyses revealed that high memorability images were better remembered than medium and low memorability images, and medium memorability images were better remembered than low memorability images (*t*s > 3.81, *p*s < .001, *d*s > .44, all post-hoc analyses were Bonferroni corrected). The main effect of the experiment was not significant, *F*(1, 47) = .08, *p* = .779, $\eta_{p}^{2}$= .002.

There was a significant interaction between experiment and memorability, *F*(1.76, 82.58) = 11.29, *p* < .001, $\eta_{p}^{2}$= .194. However, post-hoc analyses showed no significant difference between experiments across low, medium, and high memorability conditions (*t*s > 2.19, *p*s > .099, *d*s < .63, Bonferroni corrected). Across Experiments 1A and 1B, face memory accuracy (53.64% ± 12.13) was numerically lower than scene memory accuracy (63.80% ± 16.94) for high-memorability images while the opposite pattern was found for low-memorability images (face: 46.45% ± 17.93, scene: 38.00% ± 18.54). However, these differences were not statistically significant.

Experiments 1A and 1B recruited different groups of participants, face and scene stimuli had different rage of memorability score distributions (see Fig. 2A), and the overall memory performance was low possibly due to task difficulty. Furthermore, post-hoc analyses revealed that the effects of experiment (i.e., stimulus type) did not significantly differ across the memorability levels. Thus, this pattern of interaction between memory performance and stimulus type should be interpreted with caution.

**Experiments 2A (Korean, faces and scenes)**

When the recognition accuracy from the face and scene experiments was compared using a repeated-measures ANOVA with stimulus type and memorability as within-subject factors, the main effect of stimulus type was significant, *F*(1, 29) = 29.99, *p* < .001, $\eta_{p}^{2}$= .508. A post-hoc test showed that scene images were remembered better than face images on average, *t*(29) = 5.48, *p* < .001, *d* = .70.

The main effect of memorability was also statistically significant, F(2, 58) = 38.73, p < .001, $\eta_{p}^{2}$= .572. Bonferroni corrected post-hoc analyses revealed that high memorability images were better remembered than medium and low memorability images, and medium memorability images were better remembered than low memorability images, *t*s > 3.44, *p*s < .003, *d*s > .40.

Further, the interaction between stimulus type and memorability was significant, *F*(1.67, 48.44) = 6.13, *p* = .007, $\eta_{p}^{2}$= .173. Bonferroni corrected post-hoc analyses revealed that scene images showed higher recognition accuracy than face images in the medium and high memorability conditions, *t*s > 5.21, *p*s < .003, *d*s > .95 (medium memorability scenes vs. faces: 87.6% ± 11.42 vs. 73.33% ± 14.64, high memorability scenes vs. faces: 94.44% ± 8.59 vs. 78.44% ± 17.30). However, memory accuracies for low memorability faces and scenes did not differ, *t*(29) = .32, *p* > .99, *d* = .06 (low memorability scenes vs. faces: 72.00% ± 20.96 vs. 70.66% ± 14.18). This pattern of interaction could simply reflect that the memorability distributions of the face and scene stimuli were different (Fig. 4A). Also, as was in Experiments 1A and 1B, these results are consistent with previous findings that showed lower memory accuracy for faces than scenes (Bainbridge, 2019).

**Experiment 2B (US, faces and scenes)**

When face and scene memory tasks were compared, the main effect of stimulus type was again significant, *F*(1, 34) = 11.09, *p* = .002, $\eta_{p}^{2}$= .246. A post-hoc test showed that scene images were remembered better than face images on average, *t*(34) = 3.33, *p* = .002, *d* = .42.

The main effect of stimulus memorability was also statistically significant, *F*(1.42, 48.37) = 44.72, *p* < .001, $\eta_{p}^{2}$= .568. Bonferroni corrected post-hoc analyses revealed that high memorability images were better remembered than medium and low memorability images, *t*s > 6.77, *p*s < .001, *d*s > .84. The average memory accuracy for medium memorability images was marginally greater than that for low memorability images, *t*(34) = 2.33, *p* = .068, *d* = .29.

In addition, stimulus type and memorability showed a significant interaction, *F*(1.49, 50.76) = 22.39, *p* < .001, $\eta_{p}^{2}$= .397. Bonferroni corrected post-hoc analyses revealed that scene images showed higher recognition accuracy than face images in the medium and high memorability conditions, *t*s > 5.20, *p*s < .003, *d*s > .88 (medium memorability scenes vs. faces: 84.28% ± 10.51 vs. 70.42% ± 18.20, high memorability scenes vs. faces: 89.71% ± 13.36 vs. 74.66% ± 16.78). However, memory accuracies for low memorability face and scene images did not differ, *t*(34) = 1.94, *p* = .183, *d* = .33 (low memorability scenes vs. faces: 59.42% ± 22.07 vs. 67.23% ± 16.69). These results mirror the findings of Experiment 2A, suggesting that Korean participants and the US MTurk workers showed a similar pattern of results.

|  | N (# of females) | Mean age (SD) | Cultural group |
| --- | --- | --- | --- |
| Experiment 1A | 24 (16) | 22.00 ± 2.10 | South Korea |
| Experiment 1B | 25 (15) | 21.96 ± 2.24 | South Korea |
| Experiment 2A | 30 (24) | 21.16 ± 2.09 | South Korea |
| Experiment 2B | 35 (N/A) | N/A | US (MTurk) |

**Supplementary Table 1.** Description of the participants and experiment.

**References**

Bainbridge, W. A. *Memorability: How what we see influences what we remember*. 1 edn, (Elsevier Inc., 2019).
